# Inflammatory Biomarkers as Prognostic Indicators for Intracranial Aneurysm Recurrence After Stent‐Assisted Coil Embolization

**DOI:** 10.1002/iid3.70240

**Published:** 2025-07-29

**Authors:** Jie Wei, Jinghui Lin, Junjun Zhang, Zifeng Dai, Yiyong Zeng, Xianru Li, Yong Li, Jianfei Zhang, Zhiqing Lin, Shengjun Zhou

**Affiliations:** ^1^ Department of Neurosurgery The First Affiliated Hospital of Ningbo University Ningbo China

**Keywords:** cytokines, interleukins, prediction model, stent‐assisted coil embolization, unruptured intracranial aneurysms

## Abstract

**Background:**

The reappearance of intracranial aneurysms (IAs) after undergoing stent‐assisted coil embolization (SACE) is a significant issue in clinical practice. In this study, we analyzed blood regulatory T‐cell counts and plasma cytokine levels to assess the extent of systemic inflammation and investigate their potential association with the recurrence of IAs undergoing SACE.

**Methods:**

A total of 189 individuals with 220 unruptured IAs were included in a retrospective study, with participants categorized into groups of occlusion and recurrence according to the Raymond–Roy Scale. Initially, a univariate analysis was used to identify distinctions among clinical data, morphological parameters, and preoperative plasma cytokine levels. A logistic regression model was built using variables with a significance level of *p* < 0.05, and the specificity and sensitivity of the chosen parameters were assessed through graphical and statistical analysis using receiver operating characteristic (ROC) curve techniques.

**Results:**

In the group with recurrence, the plasma concentrations of IL‐2, IL‐10, IL‐17, and IFN‐γ were notably elevated compared to the occlusion group. Based on binary logistic regression analysis, it was found that the levels of IL‐10 (odds ratio = 1.24, 95% CI = 1.06–1.46, *p* = 0.008), IL‐17 (odds ratio = 1.45, 95% CI = 1.17–1.82, *p* < 0.001), and INF‐γ (odds ratio = 1.28, 95% CI = 1.07–1.54, *p* = 0.007) were determined to be crucial independent indicators for the recurrence of IAs. The highest predictive accuracy recurrence risk, with an area under the curve of 0.761, was achieved through the combination of IL‐2, IL‐10, IL‐17, and INF‐γ.

**Conclusions:**

Findings reveal indicate that elevated levels of plasma IL‐2, IL‐10, IL‐17, and IFN‐γ are consistently present in recurrent IAs, implying that the initial inflammatory levels in the body are a major contributor to the recurrence of IAs following SACE. The combination of IL‐2, IL‐10, IL‐17, and IFN‐γ may assist in predicting the likelihood of recurrence in IAs following SACE.

## Graphical abstract

The study found that elevated plasma levels of IL‐2, IL‐10, IL‐17, and IFN‐γ are significantly associated with the recurrence of intracranial aneurysms after stent‐assisted coil embolization. These cytokines serve as independent predictors for recurrence, suggesting that systemic inflammation plays a crucial role in the process.

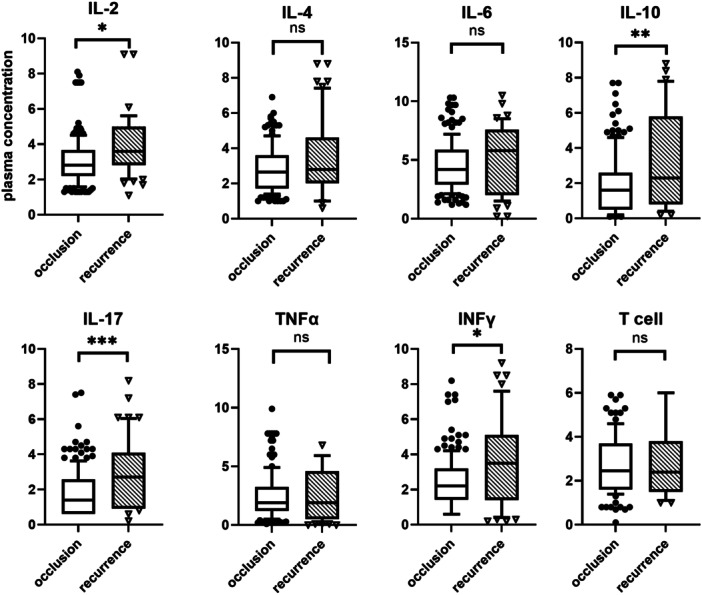

## 1 Introduction

Overall, intracranial aneurysms (IAs) continue to be a significant issue for public health, affecting approximately 0.4%–3% of the population [1]. In the management of IAs, endovascular treatment (EVT) has been established as the primary treatment option, while stent‐assisted coil embolization (SACE) has also gained considerable traction [2]. Despite clear clinical evidence demonstrating better outcomes with EVT over surgical clipping for IAs, one of the major drawbacks of EVT is the possibility of recurrence, with an estimated 6.1%–33.6% recurrence rate for IAs after EVT [3, 4]. When aneurysms recur, patients are vulnerable to re‐rupture and subsequent re‐bleeding. Consequently, assessing the risk of IA recurrence is critically important in clinical practice.

There have been numerous studies exploring the factors associated with IA recurrence. There have been numerous risk factors associated with recanalization following coil embolization, including factors like ruptured aneurysms, large dimensions, wide necks, locations in the posterior circulation, and low ratios of embolization volume [5, 6]. To predict recurrence risk following aneurysm recanalization, the Aneurysm Recanalization Stratification Scale (ARSS) has been developed [7]. The ARSS has yet to gain widespread acceptance in clinical practice, however. Utilizing computational fluid dynamics (CFD) is a successful approach to assess the hemodynamic impacts of aneurysm recurrence [8, 9, 10]. Studies suggest that the postembolization remnant neck area may have high wall shear stress (WSS) that contributes to canalization. However, the exact mechanistic relationship between these two factors is still uncertain [9, 10].

The formation and development of IA is believed to be influenced by multiple factors, such as growth factors, cytokines, chemokines, matrix metalloproteinases, and interleukins [11, 12, 13]. Furthermore, the abnormal restructuring of vessel walls may also be influenced by immune cells such as monocytes and macrophages, neutrophils, T lymphocytes, and natural killer cells (NKs) [14, 15, 16]. The enhancement of the aneurysmal wall (AWE) identified through high‐resolution magnetic resonance imaging (HR‐MRI) correlates well with inflammation within the aneurysmal wall, according to a pathological study [17]. The immune system is also activated by inflammation in the aneurysmal wall, including neutrophils, macrophages, and other inflammatory cells infiltrating the wall [18]. Interleukin‐10 and neutrophil to lymphocyte ratio (NLR) have been associated with AWE based on HR‐MRI results [19]. As AWE has emerged as a radiographic biomarker of IA instability, suggesting that inflammatory processes may be expressed in the aneurysmal wall by a blood inflammatory index. However, there has no conducted on the relationship between Pro‐inflammatory cytokines and unruptured intracranial aneurysms (UIAs).

The purpose of this study was to analyze blood regulatory T‐cell counts and plasma cytokine levels to assess the levels of systemic inflammation and investigate their potential association with the recurrence of IAs undergoing SACE.

## 2 Methods

### 2.1 Ethical Statement

The Independent Ethics Committee of The First Affiliated Hospital of Ningbo University reviewed and granted approval for this study (2023‐199RS). The reporting of this study followed the STROBE guideline (Strengthening the Reporting of Observational Studies in Epidemiology).

### 2.2 Patient Selection

A total of 436 consecutive patients with unruptured IAs were treated at our institution between January 2021 and January 2023.

Included criteria were as follows: (1) patients with unruptured saccular aneurysm; (2) patients aged 18–75 years; (3) patients treated with stent‐assisted coil embolization; and (4) patients with complete clinical data.

The following patients were excluded from the study: (1) refused to participate in the study; (2) lack of laboratory data; (3) immediate postoperative angiography demonstrated incomplete aneurysm occlusion; (4) presence of fusiform, traumatic, dissecting, and infectious IAs; (5) irregular use of antiplatelet drugs (aspirin and ticagrelor); (6) presence of chronic inflammatory diseases as well as acute infections.

### 2.3 Data Collection

Data on each patient's clinical condition were collected, including their age, sex, hypertension history, diabetes, and smoking. The parameters of morphological analysis were calculated depending on the location of the aneurysm and 3D digital subtraction angiography (DSA). In the morning of the second day after admission, following an overnight fast, blood samples were collected. Plasma cytokine levels and regulatory T‐cell counts were assessed during routine laboratory tests before the procedure. As a result of IA occlusion, the DSA outcomes were categorized according to Raymond‐Roy, with class I indicating complete occlusion, class II indicating a neck remnant, and class III indicating a residual aneurysm. Finally, the Aneurysm follow‐up at 6 months postsurgery revealed a class II or III is defined as a recurrence group, while a class I is defined as the occlusion group.

### 2.4 Statistical Analysis

SPSS 25.0 (IBM Corp, Armonk, NY) and GraphPad software (version 9.0, GraphPad Software Inc., San Diego, CA, USA) were used to analyze and visualize the obtained data. We expressed quantitative variables as median, interquartile range (IQR), or range, as well as number of patients (%) as appropriate. Statistical significance was determined using a *p* < 0.05 (two‐sided). Among cross‐tabulation and measured data, we employed the chi‐square test and the Mann–Whitney *U* test, respectively, to examine differences in baseline, morphological, and plasma cytokine levels between two groups. Independent variables were tested for collinearity, but no collinearity was detected. With a logistic regression model, the independent predictors of IA recurrence risk were assessed based on the analysis of multivariable variables with *p* < 0.05. Graphical and statistical analysis of receiver operating characteristic (ROC) curves was conducted on selected parameters to assess their specificity and sensitivity.

## 3 Results

### 3.1 General Characteristics Between Occlusion and Recurrence IAs

A total of 189 patients with 220 UIAs were enrolled in the study. Among these, 168 aneurysms exhibited complete occlusion during the 6‐month postoperative DSA follow‐up (occlusion group; Raymond I), while 51 aneurysms displayed recurrence in either the neck or sac during the 6‐month postoperative follow‐up (recurrence group; Raymond II, III).

As shown in Table 1, there were no statistically significant differences in baseline parameters (age, sex, hypertension, diabetes, drinking, smoking, IA location, IA size, IA height, and IA neck) between the two groups (Table 1).

**Table 1:** Baseline, morphological, and plasma cytokines characteristics in two groups.

Variable	Occlusion group	Recurrence group	Statistical methods	*p* value
	Raymond I (*n* = 168)	Raymond II, III (*n* = 51)		
Age (years)	60.2 ± 0.8	61.2 ± 1.3	*t*‐test	0.479
Female	120 (71.4%)	36 (70.6%)	Chi‐squared	0.908
Hypertension	99 (58.9%)	36 (70.6%)	Chi‐squared	0.134
Diabetes	22 (11.9%)	9 (17.6%)	Chi‐squared	0.414
Smoking	34 (20.2%)	12 (23.5%)	Chi‐squared	0.613
Drinking	48 (28.6%)	13 (25.4%)	Chi‐squared	0.667
Location			Chi‐squared	0.078
ICA	60 (35.7%)	9 (17.6%)		
AcomA/ACA	13 (7.7%)	3 (5.9%)		
MCA	36 (21.4%)	14 (27.5%)		
PcomA/PC	59 (35.1%)	25 (49.0%)		
Size (mm)	3 (2.23–4.30)	3.5 (2.8–4.5)	Mann–Whitney *U*	0.057
Height (mm)	2.7 (2.2–3.6)	3.4 (2.4–4.3)	Mann–Whitney *U*	0.053
Aneurysm neck (mm)	2.4 (2.0–3.0)	2.6 (2.0–3.4)	Mann–Whitney *U*	0.455
IL‐2 (pg/mL)	2.8 (2.2–3.67)	3.6 (2.8–5.0)	Mann–Whitney *U*	0.001
IL‐4 (pg/mL)	2.65 (1.7–3.6)	2.8 (2.0–4.6)	Mann–Whitney *U*	0.059
IL‐6 (pg/mL)	4.2 (2.9–5.87)	5.8 (2.0–7.6)	Mann–Whitney *U*	0.203
IL‐10 (pg/mL)	1.60 (0.5–2.6)	2.3 (0.8–5.8)	Mann–Whitney *U*	0.002
IL‐17 (pg/mL)	1.40 (0.6–2.58)	2.7 (0.9–4.1)	Mann–Whitney *U*	< 0.001
TNF‐α (pg/mL)	1.90 (1.20–3.25)	1.91 (0.5–4.6)	Mann–Whitney *U*	0.424
INF‐γ (pg/mL)	2.2 (1.43–3.20)	3.50 (1.4–5.1)	Mann–Whitney *U*	0.005
T reg (%)	2.45 (1.6–3.7)	2.4 (1.5–3.8)	Mann–Whitney *U*	0.964

Abbreviations: ACA, anterior cerebral artery; AcomA, anterior communicating artery; ICA, internal carotid artery; IL‐2, interleukin‐2; IL‐4, interleukin‐4; IL‐6, interleukin‐6; IL‐10, interleukin‐10; IL‐17, interleukin‐17; INF‐γ, interferon‐γ; MCA, middle cerebral artery; PC, posterior circulation; PcomA, posterior communicating artery; TNF‐α, tumor necrosis factor‐α; T‐reg, (CD3/CD4/CD25/CD127) regulatory T‐cells.

### 3.2 Plasma Levels of Pro‐Inflammatory Cytokines Between Occlusion and Recurrence IAs

**Figure 1:**
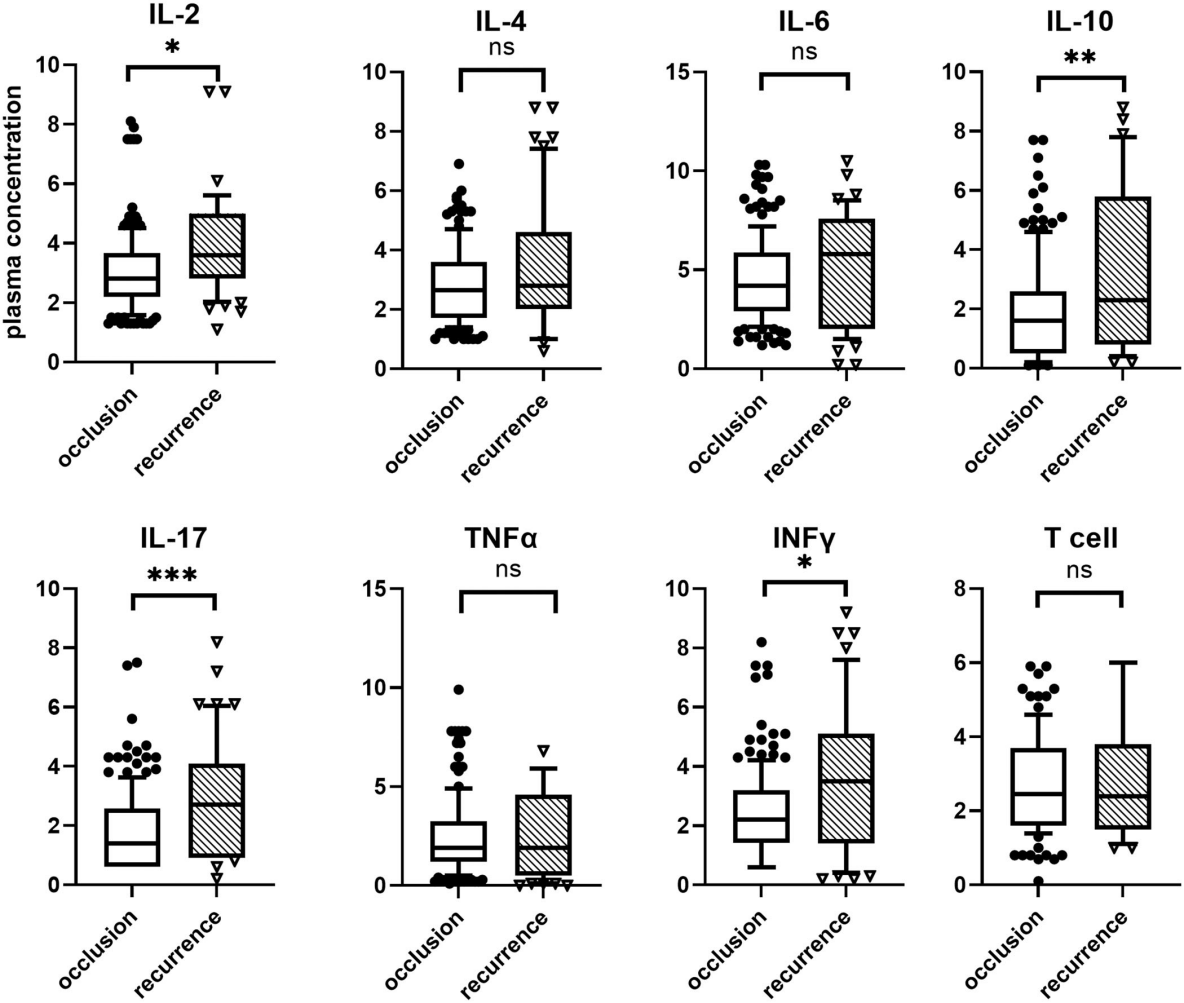
The results of univariate analysis between two groups. *p ≤ 0.05, **p ≤ 0.01, ***p ≤ 0.001. IL‐2, interleukin‐2; IL‐4, interleukin‐4; IL‐6, interleukin‐6; IL‐10, interleukin‐10; IL‐17, interleukin‐17; INF‐γ, interferon‐γ; TNF‐α, tumor necrosis factor‐α.

Plasma levels of IL‐2, IL‐10, IL‐17, and IFN‐γ were notably elevated in the recurrence group compared to the control group, as shown in Table 1 and Figure 1.

Results of the binary logistic regression analysis, the plasm concentration of IL‐10 (odds ratio = 1.24, 95% CI = 1.06–1.46, *p* = 0.008), IL‐17 (odds ratio = 1.45, 95% CI = 1.17–1.82, *p* < 0.001), and INF‐γ (odds ratio = 1.28, 95% CI = 1.07–1.54, *p* = 0.007) were independent predictive factors for IAs recurrence (Table 2).

**Table 2:** Multivariate analysis of variables that associated with IA recurrence.

Variable	Odds ratio	95% CI	*p* value
IL‐2	1.17	0.92–1.48	0.19
IL‐10	1.24	1.06–1.46	0.008
IL‐17	1.45	1.17–1.82	< 0.001
INF‐γ	1.28	1.07–1.54	0.007

Abbreviations: IA, intracranial aneurysm; interleukin‐17; IL‐2, interleukin‐2; IL‐10, interleukin‐10; IL‐17; INF‐γ, interferon‐γ.

### 3.3 Mathematical Model for Predicting IA Recurrence Risk

**Figure 2:**
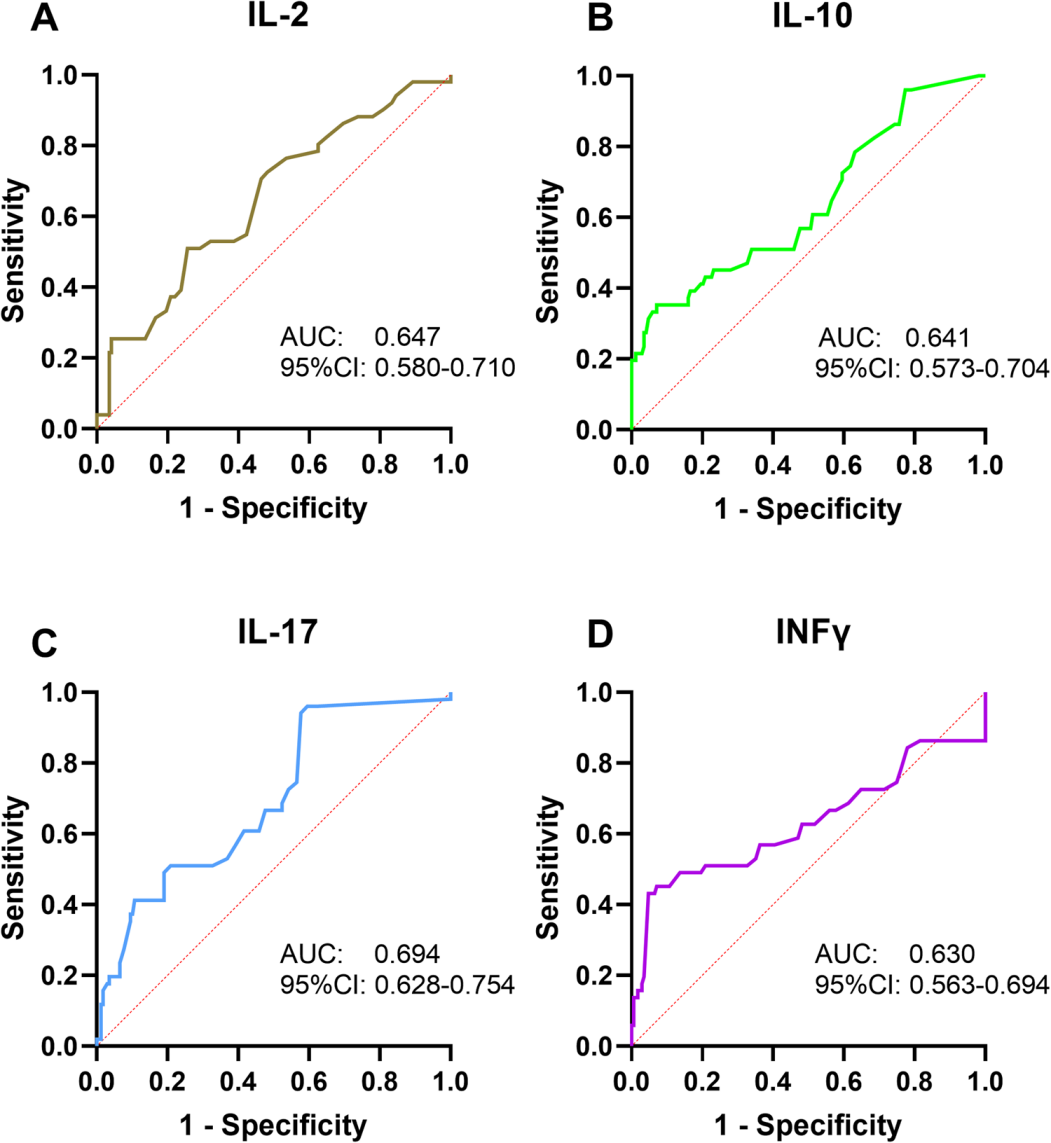
ROC curve of plasma pro‐inflammatory cytokines in prediction IA recurrence. (A) ROC curve of IL‐2; (B) ROC curve of IL‐10; (C) ROC curve of IL‐17; (D) ROC curve of IFN‐γ. AUC, area under the curve; IL‐2, interleukin‐2; IL‐10, interleukin‐10; IL‐17, interleukin‐17; INF‐γ, interferon γ; ROC, receiver operating characteristic.

**Figure 3:**
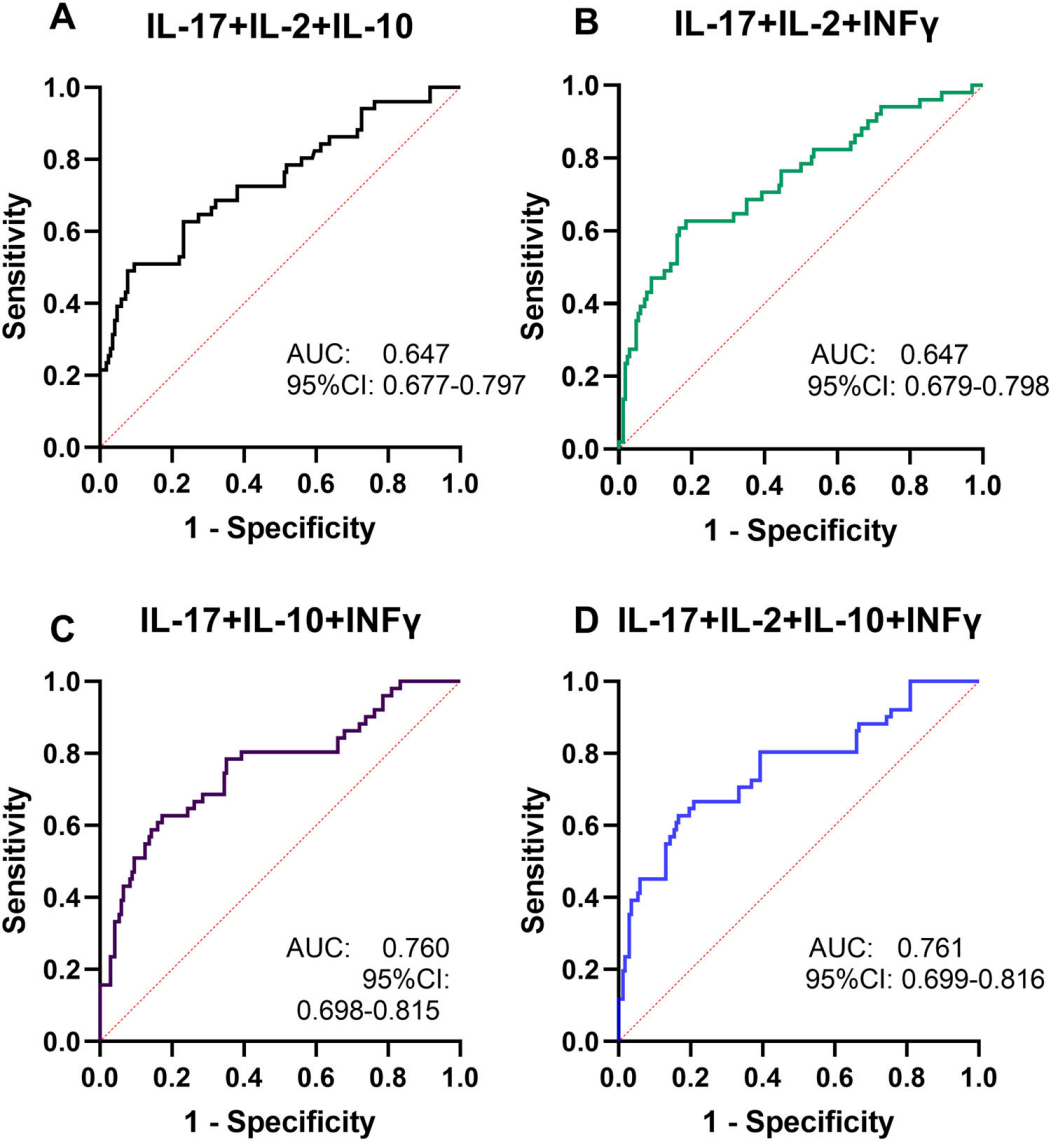
ROC curve for the combination of pro‐inflammatory cytokines in prediction IA recurrence. (A) ROC curve of IL‐17, IL‐2 and IL‐10; (B) ROC curve of IL‐17, IL‐2 and INF‐γ; (C) ROC curve of IL‐17, IL‐10 and INF‐γ; (D) ROC curve of IL‐17, IL‐2, IL‐10 and INF‐γ. IL‐2, interleukin‐2; IL‐10, interleukin‐10; IL‐17, interleukin‐17; INF‐γ, interferon γ; ROC, receiver operating characteristic.

As shown in Figure 3 and Table 2, ROC analysis indicated the potential value of the plasma pro‐inflammatory cytokines in predicting IA recurrence risk. The area under the curve (AUC) values of IL‐2, IL‐10, IL‐17, and INF‐γ for predicting the risk of IAs recurrence were 0.647 (*p* < 0.001; 95% CI = 0.580–0.710), 0.641 (*p* = 0.0025; 95% CI = 0.573–0.704), 0.694 (*p* < 0.0001; 95% CI = 0.628–0.754), and 0.725 (*p* = 0.0158; 95% CI = 0.563–0.694), respectively (Table 3, Figure 2). The optimal cutoff values for the above parameters were 3.5, 4.7, 0.7, and 4.9, respectively. The IL‐17 achieved the highest AUC (0.694) among the single parameters.

**Table 3:** Statistical data of receiver‐operating characteristics curve comparisons of different parameters in predicting IA recurrence.

	AUC (95% CI)	*p* value	Cut‐off value		Youden index	Sensi	Speci
IL‐2	0.647 (0.580–0.710)	0.0009	> 3.5		0.2539	50.98	74.40
IL‐10	0.641 (0.573–0.704)	0.0025	> 4.7		0.2815	35.29	92.86
IL‐17	0.694 (0.628–0.754)	< 0.0001	> 0.7		0.3655	96.08	40.48
INF‐γ	0.630 (0.563–0.694)	0.0158	> 4.9		0.3838	43.14	95.24

Abbreviations: IA, intracranial aneurysm; interleukin‐17; IL‐2, interleukin‐2; IL‐10, interleukin‐10; IL‐17; INF‐γ, interferon‐γ.

ROC analysis of the combined test results for pro‐inflammatory cytokines revealed an AUC of 0.71 (*p* < 0.0001; 95% CI = 0.889–0.974; Figure 3A and Table 3) for the combination of IL‐17, IL‐2, and IL‐10, an AUC of 0.742 (*p* < 0.0001; 95% CI = 0.679–0.798; Figure 3B and Table 4) for combination of IL‐17, IL‐2, and INF‐γ, an AUC of 0.761 (*p* < 0.0001; 95% CI = 0.698–0.815; Figure 3C and Table 4) for combination of IL‐17, IL‐10, and INF‐γ, an AUC of 0.760 (*p* < 0.0001; 95% CI = 0.699–0.816; Figure 3B and Table 4) for combination of IL‐17, IL‐2, and INF‐γ, respectively. The AUC for the combination of IL‐17, IL‐2, IL‐10, and IFN‐γ was 0.761 (*p* < 0.0001; 95% CI = 0.699–0.816; Figure 3D and Table 4) in discriminating IAs with high recurrence risk from post‐SACE IAs.

**Table 4:** Statistical data of receiver‐operating characteristics curve comparisons of combination parameters in predicting IA recurrence.

	AUC (95% CI)	*p* value	Youden index	Sensi	Speci
IL‐17 + IL‐2 + IL‐10	0.740 (0.677–0.797)	< 0.0001	0.4146	50.98	90.48
IL‐17 + IL‐2 + INF‐γ	0.742 (0.679–0.798)	< 0.0001	0.4429	62.75	81.55
IL‐17 + IL‐10 + INF‐γ	0.760 (0.698–0.815)	< 0.0001	0.4548	62.75	82.74
IL‐17 + IL‐2 + IL‐10 + INF‐γ	0.761 (0.699–0.816)	< 0.0001	0.4608	62.75	83.33

Abbreviations: IA, intracranial aneurysm; interleukin‐17; IL‐2, interleukin‐2; IL‐10, interleukin‐10; IL‐17; INF‐γ, interferon‐γ.

## 4 Discussion

The purpose of this study was to measure the plasma concentrations of pro‐inflammatory cytokines in patients with IAs who had undergone SACE. Our analysis revealed that, in comparison to occluded UIAs, post‐SACE UIAs with a higher recurrence risk exhibited higher plasma concentrations of IL‐2, IL‐10, IL‐17, and IFN‐γ. Additionally, the concentrations of IL‐10, IL‐17, and INF‐γ were identified as independent predictive factors for IA recurrence.

A complex process, the development of IA relies on the interaction between a number of molecules, such as chemokines, growth factors, interleukins, cytokines, and matrix metalloproteinases [20]. A modification of the arterial wall, ostensibly correlated with inflammatory responses and repair mechanisms, is causatively linked to the initiation and subsequent rupture of IA [20]. Some studies suggest that aneurysm growth is associated with inflammation‐mediated remodeling triggered by blood flow. Endothelial cells sense blood flow and WSS to influence the remodeling of collagen fibers in aneurysm walls [21]. Meng et al. used surgically created high‐flow bifurcations to demonstrate changes prone to inducing aneurysm formation, such as internal elastic lamina disruption and smooth muscle cell layer degeneration, occurring in areas exposed to high WSS and positive WSS gradients [22]. The colocalization of high WSS with wall stretch leads to the amplification of MCP‐1 expression and macrophage infiltration, ultimately triggering the development of IAs [23].

SACE has been widely recognized as an effective and minimally invasive alternative to surgical clipping for occluding ruptured and unruptured IAs [24]. Nevertheless, subsequent investigations have revealed instances of coil compaction and recanalization or regrowth of the treated aneurysm in some cases [25]. The underlying biological principle of coiling in the treatment of aneurysms is the induction of intra‐aneurysmal thrombosis. In cases of successful and complete aneurysm healing, the initially disorganized intraluminal hematoma evolves into granulation tissue, ultimately maturing into organized fibrosis [26]. Histopathological studies in both human and preclinical settings have unveiled a growth pattern originating from the aneurysm wall. During this progression, the endothelial cell lining expands centripetally over the granulation tissue toward the aneurysm neck's center [27, 28]. Cell migration from the artery wall and from the aneurysm wall is necessary for IA healing after SACE in a rat saccular sidewall model [29]. In previous studies, it has been found that a reduction in mural cells results in chronic inflammation and inadequate healing, ultimately resulting in aneurysm regrowth and rupture [26, 30]. SACE has shown to be a highly effective treatment for UIA patients, so it seems particularly important to evaluate human plasma cytokines levels in these patients.

A subset of CD34+ hematopoietic progenitor cells, called endothelial progenitor cells (EPCs), differentiates into endothelial cells in vitro. In the regeneration of damaged or dysfunctional endothelial cells, EPCs play a crucial role in maintaining vascular integrity [31]. In particular, recent studies have shown that EPCs migrate to curved regions, enhancing endothelialization [32]. We hypothesized that human inflammation levels would affect IA healing after SACE treatment, and plasma cytokine concentration reflects the level of inflammation in the human body. UIAs with higher plasma cytokine concentration will impede vascular endothelial regeneration, thereby leading to IA recurrence. Therefore, we anticipate a correlation between plasma cytokine and the risk of IA rupture.

Researchers have extensively studied the role of proteins in IA growth, rupture, and formation. Studies on IA using animal models have demonstrated elevated mRNA and protein expression of interleukin‐1β (IL‐1β), interleukin‐8 (IL‐8), tumor necrosis factor‐α (TNF‐α), and monocyte chemoattractant protein‐1 (MCP‐1) within endothelial cells [15, 16, 33]. These investigations suggest the involvement of the aforementioned cytokines in the pathomechanism underlying aneurysm formation. While definitively establishing whether these cytokines are the root cause or a consequence of aneurysm development is challenging, it is certain that, in conjunction with immunocompetent cells, they contribute to the pathological remodeling of cerebral vessels. Pro‐inflammatory cytokines such as IL‐1β, IL‐6, IL‐8, TNF‐α, and IFN‐γ are primarily released by macrophages, exerting a significant influence on the initiation of IA [34, 35]. The results of our research draw attention to the cytokines IL‐2, IL‐4, IL‐6, IL‐17, TNF‐α, IFN‐γ, and regulatory T‐cell counts, whose role in the recurrence of IA has so far, not been widely studied.

We developed a mathematical model to predict recurrence risk of IAs using binary logistic regression analysis. Our extensive forecasting model, which includes IL‐2, IL‐10, IL‐17, and IFN‐γ, achieved a sensitivity of 0.6275 and a specificity of 0.8333. As a result of our findings, we can further improve clinical recurrence prediction accuracy by combining plasma pro‐inflammatory cytokines. In spite of the model's favorable specificity, there is room for improvement in terms of its sensitivity. A prospective study with a larger number of patients is needed to confirm the findings, as well as animal experiments to confirm the role of pro‐inflammatory cytokines in the recurrence of IA.

## 5 Limitation

There are several limitations to this study. First, the retrospective design and the relatively small sample size may introduce bias into the data collection and analysis. Second, the exclusive focus on preoperative plasma cytokine levels may not fully reflect the long‐term immune status of the patients. Additionally, the study did not investigate the mechanisms by which cytokine levels might influence aneurysm recurrence following SACE, Lastly, the clinical benefits of measuring plasma cytokine levels still require larger‐scale studies.

## 6 Conclusions

High levels of plasma IL‐2, IL‐10, IL‐17, and IFN‐γ were consistently observed in recurrent IAs, suggesting that baseline inflammatory levels in the human body play a significant role in the recurrence of IAs after SACE. Combining IL‐2, IL‐10, IL‐17, and IFN‐γ could help predicting the recurrence risk for IAs after SACE.

## Author Contributions

**Jie Wei:** conceptualization, data curation, methodology, writing – original draft, writing – review and editing. **Jinghui Lin:** conceptualization, funding acquisition, visualization, writing – review and editing. **Junjun Zhang:** data curation, methodology. **Zifeng Dai:** methodology, software. **Yiyong Zeng:** data curation. **Xianru Li:** data curation. **Yong Li:** data curation. **Jianfei Zhang:** data curation, validation, visualization. **Zhiqing Lin:** validation, visualization. **Shengjun Zhou:** resources, software, supervision, visualization, writing – review and editing.

## Supporting information

STROBE Checklist.

## Data Availability

The authors have nothing to report.
